# Navigated oblique lumbar interbody fusion for adult spinal deformity

**DOI:** 10.3171/2020.1.FocusVid.19700

**Published:** 2020-01-01

**Authors:** Chih-Chang Chang, Joshua Rivera, Brenton Pennicooke, Dean Chou, Praveen V. Mummaneni

**Affiliations:** 1 Department of Neurosurgery, University of California, San Francisco, California;; 2 Department of Neurosurgery, Neurological Institute, Taipei Veterans General Hospital, Taipei;; 3 School of Medicine, National Yang-Ming University, Taipei, Taiwan; and; 4 University of California, San Francisco, California

**Keywords:** adult spinal deformity, minimally invasive surgery, OLIF, oblique lumbar interbody fusion, prepsoas, video

## Abstract

Adult spinal deformity (ASD) is an increasing disease entity as the population ages. An emerging minimally invasive surgery (MIS) option for the treatment of ASD is the oblique lumbar interbody fusion (OLIF), which allows indirect foraminal decompression of stenosis as well as segmental deformity correction (DiGiorgio et al., 2017). The authors utilize computer-assisted navigation with OLIF to reduce radiation exposure and improve time efficiency. The authors present a video of navigated oblique lumbar interbody fusion at L3–5 followed by open posterior screw-rod fixation.

The video can be found here: https://youtu.be/zKDT7PhMYf8.

## Transcript

**0:20** This is the video of the navigated oblique lumbar interbody fusion. This is the patient history. The patient presents with chief complaint of back pain with progressive lower extremity numbness for 2 years. These are the radiographic images. On the left, you can see the CT scan demonstrating a lateral listhesis of L3–4. On the right is the MRI images demonstrating the axial cuts through L2–3, L3–4, and L4–5. Here you can see a long-standing x-rays demonstrating the patient’s deformity and on the right are showing the spinopelvic parameters. And oblique lumbar interbody fusion is planned for three reasons. First, to help correct the coronal deformity. Second, to enhance arthrodesis. And third, to address a lateral listhesis that is seen on L3–4.

**1:19** The patient is positioned in right lateral decubitus position. Using navigation, the midpoint of the vertebral body is identified. A ruler is used to measure 5 cm anterior to the midpoint. This is how the incision is planned.

**1:36** After opening the skin and the fascia, the muscles are dissected bluntly. The three muscular layers of external oblique, internal oblique, and transversus abdominis are bluntly dissected until the retroperitoneal fat is reached. Once retroperitoneal fat has been reached, manual dissection is then used to create a pocket in the abdominal cavity for the surgery.

**2:06** Light retractor is then used to dissect to the psoas muscle anterior to the spine. Navigation confirms position. And after confirming positioning, the dilators are placed in order to place the retractor. Here you can see how the table-mounted retractors positioned and placed with oblique angulation toward posterior aspect of the spine. The width of the minimally invasive tubular retractor is 22 mm, and this can be expanded as necessary.

**2:38** This is the visualization that is achieved after the retractor has been placed and light sources are also placed. After that is being performed, the disc is incised and, using rongeurs, the disc material is subsequently removed. This can be done with rongeurs and combination of curette to loosen the disc and to remove as much disc as possible. This is the visualization that is seen through the retractor.

**3:17** Curettes are then used to loosen the disc material. And afterward, pituitary rongeurs are used to remove the fragments. After these has been performed, a navigated Cobb is then inserted in an oblique manner. After partially going into the disc space, the Cobb is then rotated out of the retractor into a true lateral position. Thus, even though the surgery is performed with oblique approach, the interbody fusion and other instrument works toward a true lateral fashion.

**3:52** Here you can see a ring curette going down and loosening further disc material and preparing the endplates. This is the navigated endplate shaver to prepare the endplates and remove all of the cartilaginous endplates. You can see here that the instruments are placed through the retractor in oblique manner, but then subsequently rotated outward to a true lateral position.

**4:21** This is the trial being placed in a true lateral position just by the oblique approach. This is the implant has been filled with graft material for arthrodesis. Metallic slides are used in order to protect the endplates as the implant is going in. This not only protects the endplates from violation but also contains the graft material.

**4:47** The postoperative CT and x-ray demonstrate the construct.

## Acknowledgments

Dr. Chang has received a grant from Yen Tjing Ling Medical Foundation, a nongovernment, nonprofit organization.
